# Protocadherin 19 regulates axon guidance in the developing *Xenopus* retinotectal pathway

**DOI:** 10.1186/s13041-024-01130-5

**Published:** 2024-08-22

**Authors:** Jane Jung, Jugeon Park, Sihyeon Park, Chul Hoon Kim, Hosung Jung

**Affiliations:** 1 Department of Anatomy, Graduate School of Medical Science, Brain Korea 21 Project, Yonsei University College of Medicine, Seoul, 03722 Republic of Korea; 2 Department of Pharmacology, Graduate School of Medical Science, Brain Korea 21 Project, Yonsei University College of Medicine, Seoul, 03722 Republic of Korea; 3 Pohang University of Science and Technology (POSTECH), Pohang, Republic of Korea

**Keywords:** Pcdh19, Axon guidance, Midline crossing, Fasciculation, Retinal ganglion cell, Xenopus tropicalis

## Abstract

Protocadherin 19 (Pcdh19) is a homophilic cell adhesion molecule and is involved in a variety of neuronal functions. Here, we tested whether Pcdh19 has a regulatory role in axon guidance using the developing *Xenopus* retinotectal system. We performed targeted microinjections of a translation blocking antisense morpholino oligonucleotide to knock down the expression of Pcdh19 selectively in the central nervous system. Knocking down Pcdh19 expression resulted in navigational errors of retinal ganglion cell (RGC) axons specifically at the optic chiasm. Instead of projecting to the contralateral optic tectum, RGC axons in the Pcdh19-depleted embryo misprojected ipsilaterally. Although incorrectly delivered into the ipsilateral brain hemisphere, these axons correctly reached the optic tectum. These data suggest that Pcdh19 has a critical role in preventing mixing of RGC axons originating from the opposite eyes at the optic chiasm, highlighting the importance of cell adhesion in bundling of RGC axons.

**Supplementary Information:**

The online version contains supplementary material available at 10.1186/s13041-024-01130-5.

Protocadherin 19 (Pcdh19) is a member of the δ2-protocadherin family of homophilic cell adhesion molecules [1] and one of the most common human genes linked to epilepsy syndrome [2, 3]. Pcdh19 is highly expressed in the brain and believed to play roles in cell adhesion, cell migration, neurogenesis, immediate early gene expression, and synapse formation [4–9]. However, it remains unclear whether alterations in any of these processes contribute to the pathogenesis of Pcdh19-related epilepsy in human.

Retinal ganglion cells (RGCs) are the projection neurons of the vertebrate visual system, conveying information on the visual hemifield to the contralateral brain hemisphere [10]. In animals with monocular vision, such as fish and pre-metamorphic frog tadpoles, all RGC axons cross the midline at the optic chiasm and terminate at the contralateral optic tectum, whereas in animals with binocular vision, they either cross or avoid the midline. The current view is that the default route for RGC axons is contralateral and that repulsive ephrin B at the optic chiasm promotes ipsilateral routing of a subset of RGC axons expressing EphB [11, 12]. However, the mechanism by which RGC axons from opposite eyes avoid mixing at the optic chiasm and continue projecting contralaterally remains unknown. Pcdh19 is expressed in the embryonic retinal ganglion cells [13, 14], and has been proposed to play a potential role as an adhesion protein in the optic nerve fiber bundling[15]. This possibility has never been experimentally tested.

We utilized pre-metamorphic *Xenopus tropicalis* tadpoles (stage 45) as a model, where Ephrin B is not yet expressed and all RGC axons project contralaterally [11]. We aimed to investigate whether Pcdh19 plays a role in bundling RGC axons originating from each eye and preventing them from mixing with those from the opposite eye. To achieve this, we took a loss-of-function approach based on the targeted microinjections of a translating-blocking antisense morpholino (MO) oligonucleotide for *Pcdh19* mRNA to selectively knock down the expression of Pcdh19 in the central nervous system. To visualize RGC axons in the brain, we employed anterograde DiI labeling as previously described [16] (Fig. 1A).

**Fig. 1:**
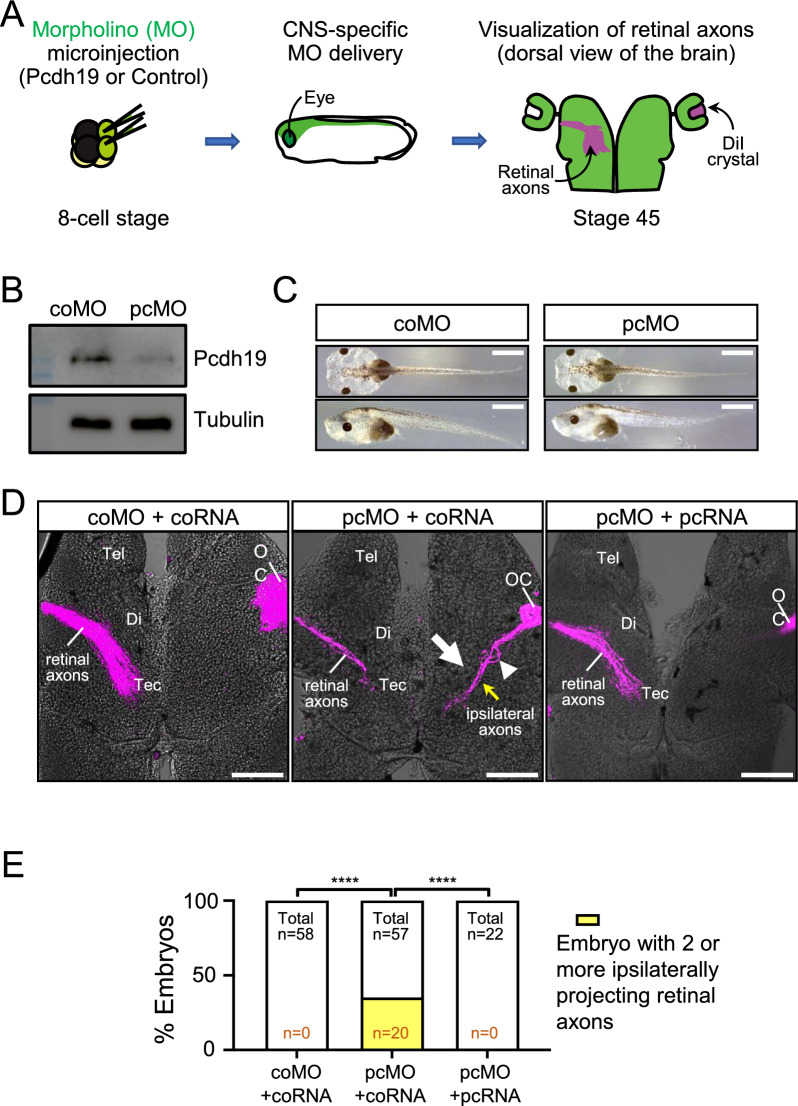
Knockdown of *Pcdh19* leads to ipsilateral misprojections of RGC axons at the optic chiasm in *Xenopus*. **A.** Experimental procedure. Morpholinos (MOs) were injected at two dorsal-animal blastomeres at the 8-cell stage, and the retinal axons were visualized by DiI at stage 45. Equal amounts of *Pcdh19* MO (pcMO) or control MO (coMO) were injected with GFP (coRNA) or mouse Pcdh19-GFP RNA (pcRNA). **B.** Efficacy of *Pcdh19* MO. Western blot from the brain lysate of pcMO or coMO-injected embryos. Tubulin was used as an internal control. **C.** Morphologies of MO-injected tadpoles. Scale bars, 1 mm. **D.** Representative confocal images of retinal axon projection in the *Xenopus* brain (Tel: Telencephalon, Di: Diencephalon, Tec: Tectum, OC: Optic chiasm). Scale bars, 100 μm. **E** The percentages of embryos having 2 or more ipsilaterally projecting axons in each group (*p* < 0.0001, Fisher’s exact test)

The injection of *Pcdh19* MO resulted in a near complete knockdown of Pcdh19 expression in the brain at stage 27 when retinal ganglion cell axons enter the brain and reach the optic chiasm [16](Fig. 1B), without any discernible change in the gross morphology of the eye, the brain and the embryo (Fig. 1C). Strikingly, *Pcdh19* MO injections led to an increase in incorrect ipsilateral projections of RGC axons. In many instances, RGC axons made navigational errors at the optic chiasm, where RGC axons from opposite eyes converge (Fig. 1D). Despite this misrouting, RGC axons followed a correct pathway, albeit on the wrong side of the brain, and terminated at the optic tectum. We quantified this phenotype by counting the number of embryos that show 2 or more ipsilaterally projecting retinal axons and found a significantly higher percentage of *Pcdh19* MO embryos with ipsilaterally misprojecting RGC axons (arrow), which were often accompanied by premature and local defasciculation (arrowhead) (Fig. 1E). This phenotype was completely rescued by expressing a MO-resistant mouse *Pcdh19* RNA, indicating that this phenotype is caused by the loss of Pcdh19 function (Fig. 1E).

Given that ipsilaterally misprojecting RGC axons in *Pcdh19* MO-injected embryos correctly reached the tectum on time, it appears that loss of Pcdh19 function does not interfere with axon growth or target entry. Among the possibilities that may have influenced RGC axons to make navigational errors in midline crossing [12], we speculate that Pcdh19, being a cell adhesion molecule, may be instead involved in RGC axon fasciculation, a process crucial for maintaining the coherence of RGC axons from the same eye at the optic chiasm, where two optic nerves decussate. In line with this idea, the loss of another cell adhesion molecules, neural cell adhesion molecule (NCAM), in mice causes ipsilateral misprojection of corticospinal tract axons at the pyramidal decussation [17]. Also in line is the notion that attenuating axon-axon interactions is a common mechanism that allows axonal reorganization following defasciculation [18]. Further investigations will be needed to reveal the Pcdh19-dependent mechanisms that regulate axon bundling and sorting at the midline more broadly. This is an important question in developmental neurobiology, as the precise control of the midline crossing of commissural axons is key to setting up the basic wiring plan of the central nervous system. In summary, these results demonstrate that Pcdh19 is required for RGC axons to remain bundled at the optic chiasm and suggest that Pcdh19 may play an important role in the axon guidance of commissural neurons.

### Supplementary Information

Additional file 1

## Data Availability

All data and materials are available upon request.

## Abbreviations

RGC: Retinal ganglion cell
MO: Morpholino
Pcdh19: Protocadherin 19
