# Supplementary material for: Protocadherin 19 regulates axon guidance in the developing Xenopus retinotectal pathway
Source: Mol Brain. 2024 Aug 22;17:58. doi: 10.1186/s13041-024-01130-5 (PMC11342623; doi:10.1186/s13041-024-01130-5)
Supplement: Supplementary file 1 — Additional file 1 [file 13041_2024_1130_MOESM1_ESM.docx]

**Materials and Methods**

**Microinjections**

*Xenopus tropicalis* frogs were purchased from Xenopus 1 Corp. (MI, USA). Embryos were generated by *in*

*vitro* fertilization and raised in 0.1X Modified Barth’s Saline (MBS) (8.8 mM sodium chloride, 100 μM

potassium chloride, 100 μM magnesium sulfate, 500 μm 4-(2-hydroxyethyl)-1- piperazineethanesulfonic

acid, 250 μM sodium bicarbonate, and 1 mM calcium chloride) at an incubator maintained at 24℃. To knockdown *Pcdh19* expression in the central nervous system, targeted micro-injections were made to two dorsal animal blastomeres at the 8-cell stage. One ng of morpholino was injected per blastomere, along with one hundred pg of RNA, which was used as a tracer (and also as a rescue construct in case of PCDH19-GFP). All injected tadpoles were screened under a fluorescence microscope to assess the correct targeting of the microinjection. pcDNA3-mPCDH19-GFP-WT, encoding the mouse PCDH19 protein fused to enhanced green fluorescent protein (EGFP) at its C-terminus, was generated in-house. Because mouse PCDH19 amino acid sequences are highly conserved in vertebrates (95% identity and 97% similarity between human and mouse, 82% identity and 91% similarity between human and *Xenopus tropicalis*), mouse PCDH19 protein was used to rescue the *Pcdh19* knockdown phenotype in *Xenopus*. Capped RNAs encoding EGFP (coRNA) or mPCDH19-GFP-WT (pcRNA) were synthesized using mMESSAGE mMACHINE SP6 Transcription Kit or mMESSAGE mMACHINE T7 Transcription Kit, respectively (Thermo Fisher Scientific, MA, USA) according to the manufacturer’s instructions. The following *Pcdh19* MO (pcMO) and control MO (coMO) were designed and supplied by GeneTools (OR, USA): *Xenopus* *Pcdh19* MO, 5’-CCCTGCTCAGCCACAACCACATAGT-3’; Control MO, 5’-CCTCTTACCTCAGTTACAATTTATA-3’.

**DiI labeling**

1,1’-Dioctadecyl-3,3,3’;’-tetramethylindocarbocyanine perchlorate (DiI, Sigma) crystals were dissolved in chloroform. Embryos were fixed in 4% paraformaldehyde in 1X phosphate buffered saline (PBS) for 2 hours at room temperature and immobilized in PBS on a dish containing solidified Sylgard (Sigma). The lens of one eye was removed, and DiI solution was filled into the cavity. DiI was allowed to diffuse approximately for 16 hours in a humidifying chamber to label retinal ganglion cell axons, after which the brain was dissected out and processed for imaging. Three independent experiments were performed before analyzing the results. Dil-labelled axons were imaged using a confocal fluorescence microscope (LSM 700, Carl Zeiss).

**Western blot analysis**

Western blot was performed using an anti-Pcdh19 (Abcam, Cambridge, UK) or anti-Tubulin (Abcam, Cambridge, UK) antibody (both at 1:1000), and the horseradish peroxidase-conjugated secondary antibodies (Abcam, Cambridge, UK). Brain lysate was extracted from stage 27 embryos, in which retinal ganglion cell axons exit the optic nerve head and enter the brain. The antibody raised against the C-terminus of the human PCDH19 protein detected *Xenopus tropicalis* Pcdh19 proteins by Western blot, as expected from high sequence identity and similarity between two species.

**Statistical Analysis**

All the analyzed data were obtained from three independent experiments, each performed with similar numbers of animals per group and per experiment. Statistical significance is indicated in the figures as *****p* < 0.0001, Fisher’s exact test.
